# Quetiapine induced ischemic colitis: about two cases

**DOI:** 10.1192/j.eurpsy.2022.1874

**Published:** 2022-09-01

**Authors:** M. Martín, M.D.C. Molina Liétor

**Affiliations:** Hospital Universitario Príncipe de Asturias, Psychiatry, Alcalá de Henares, Spain

**Keywords:** quetiapine, adverse drug reaction, drug-induced ischemic colitis, anticholinergic effect

## Abstract

**Introduction:**

Due to its anticholinergic action, antipsychotic drugs, especially phenothiazines and atypical antipsychotics, have been described as a rare cause of drug-induced ischemic colitis. We present two cases of patients that were admitted to the gastroenterology unit of a general hospital and were diagnosed of quetiapine-induced ischemic colitis.

**Objectives:**

To describe an uncommon side effect of neuroleptic treatment.

**Methods:**

Case report and literature review.

**Results:**

First patient, aged 73, with history of dysthymia, in treatment with desvenlafaxine, quetiapine, ketazolam, lorazepam, enalapril/hidroclorothiazide, omeprazole, simvastatin, tramadol/paracetamol, alendronate/colecalciferol and hidroferol, consulted in the emergency room for malaise, disorientation, haematuria, abdominal pain and changes in deposition rhythm; family members admitted frequent use of higher than prescribed doses of quetiapine and benzodiazepines. Second patient, aged 63, with history of histrionic personality disorder, in psychopharmacologic treatment with venlafaxine, quetiapine, diazepam, fentanyl, rupatadine, cinitapride, omeprazole, levosulpiride, simvastatin, fluticasone/salmeterol and celecoxib, consulted for abdominal pain and bloody diarrhoea. Colonoscopy findings in both of them were compatible with ischemic colitis. Quetiapine was withdrawn in both cases, as the main diagnostic hypothesis was quetiapine-induced ischemic colitis. The patients achieved full recovery.

**Conclusions:**

Ischemic colitis is a rare but potentially fatal adverse effect of antipsychotic drugs, with clozapine being the most reported atypical antipsychotic thought to cause it. The risk associated with quetiapine is thought to be lower given its milder anticholinergic effect. Co-prescription with other drugs with anticholinergic actions increases the risk. Clinicians should be aware of this association and the onset of constipation should alert medical staff.

**Disclosure:**

No significant relationships.

